# Enhancement in Capacitance of Ionic Type of EAP-Based Strain Sensors

**DOI:** 10.3390/s23239400

**Published:** 2023-11-25

**Authors:** Nitin Kumar Singh, Kazuto Takashima, Shyam S. Pandey

**Affiliations:** Graduate School of Life Science and Systems Engineering, Kyushu Institute of Technology, 2-4 Hibikino, Wakamatsu Campus, Kitakyushu 808-0196, Japan; ktakashima@life.kyutech.ac.jp

**Keywords:** strain sensors, electroactive polymers, DBSA-doped, PANI, SEBS rubber, enhancement in capacitance, ionic EAPs, dielectric constant

## Abstract

This paper aims to enhance the capacitance of electroactive polymer (EAP)-based strain sensors. The enhancement in capacitance was achieved by using a free-standing stretchable polymer film while introducing conducting polymer to fabricate a hybrid dielectric film with controlled conductivity. In this work, styrene-ethylene-butylene-styrene (SEBS) rubber was used as the base material, and dodecyl benzene sulfonate anion (DBSA)-doped polyaniline (PANI) was used as filler to fabricate a hybrid composite conducting film. The maleic anhydride group of the SEBS Rubber and DBSA, the anion of the polyaniline dopant, make a very stable dispersion in Toluene and form a free-standing stretchable film by solution casting. DBSA-doped polyaniline increased the conductivity and dielectric constant of the dielectric film, resulting in a significant enhancement in the capacitance of the EAP-based strain sensor. The sensor presented in this article exhibits capacitance values ranging from 24.7 to 100 µF for strain levels ranging from 0 to 100%, and sensitivity was measured 3 at 100% strain level.

## 1. Introduction

Electroactive polymers, also known as electroactive polymers (EAPs), are a type of material that can change shape or size in response to applied electrical or mechanical forces acting as external stimuli [1,2,3,4]. This property makes them useful in various applications, such as actuators, sensors, and energy-harvesting devices [5,6,7,8,9]. In generator mode, EAPs convert external mechanical energy into electrical power by undergoing deformation and redistributing charges within their structure. This process enables them to harvest energy from their surroundings, finding applications in energy-scavenging devices. As actuators, EAPs respond to applied electric fields by changing shape or size, facilitating controlled mechanical movement for tasks such as bending or twisting. This makes them valuable in the soft robotics field. Additionally, EAPs excel as sensors, detecting mechanical stimuli and converting them into electrical signals, providing critical feedback in applications such as healthcare. EAPs have many advantages over traditional materials used for these purposes, such as their light weight, low cost, and high flexibility. EAPs can be divided into two main categories: ionic EAPs and electronic types of EAPs [10,11,12,13,14]. Ionic EAPs (i-EAPs) rely on the movement of ions through the material to produce deformation, while electronic EAPs (e-EAPs) rely on the movement of electrons through the material [15,16,17,18]. The ionic class of EAPs exhibits a notable ability to produce precise and controlled actuation in response to external stimuli. Ongoing research endeavors in this area are dedicated to optimizing the ionic conductivity of these materials, with the aim of enhancing their responsiveness to stimuli. Conductive polymers and ionic polymer-metal composites (IPMCs) are common examples of ionic electroactive polymers (i-EAPs), while dielectric elastomers (DEs) and piezoelectric materials such as polyvinylidene fluoride (PVDF) are a few examples of electronic electroactive polymers (e-EAPs). EAPs have the potential to be used in a wide range of applications, including actuators for robotics, medical devices, and sensors for detecting changes in pressure, force, and strain, etc. [19,20,21,22,23]. In the recent past, it has been observed that elastomer-based strain sensors attracted researchers’ attention due to their wide applications in human activity monitoring, human-machine interface (HMI), rehabilitation, health monitoring, exoskeletons, assistive technology, augmented/virtual reality, soft robotics, and so on [24,25,26,27,28]. When these polymers are sandwiched between the two elastic electrodes, the whole system can be treated as an artificial muscle. The variable load applied to this artificial muscle changes the resistance and capacitance, which can be utilized further in detecting mechanical quantities making the basis of sensor technology [29,30]. However, resistance exhibits non-linear behavior at large deformations. As a result, it becomes challenging to calibrate mechanical quantities into electrical quantities due to this non-linearity. Therefore, researchers are currently focusing on utilizing EAP-based capacitive sensors for various applications [31,32,33]. The work in this field is in its initial phase and is evolving. Only a few research articles related to electroactive polymer-based strain sensors have been reported so far. The majority of the reported sensors exhibit a small value of capacitance, which is a drawback for their application in real-life scenarios.

Cholleti et al. presented a capacitive EAP strain sensor using a composite of barium titanate and silicone elastomer [34]. Filippidou et al. proposed a flexible capacitive EAP strain sensor based on polydimethylsiloxane (PDMS) and graphene [35]. Nitin et al. reported a fabric-based sensor that also shows capacitance in the range of pF [36]; fabric also limits the sensor’s elasticity. All the above-mentioned sensors show capacitance in the range of pF, and this small value of capacitance limits the use of sensors in a wide range of applications [37,38].

Existing elastic sensors typically have low values of both capacitance and elasticity, resulting in low sensitivity. This limitation restricts their practical application in wearables. Therefore, in this paper, we present a sensor that demonstrates high capacitance and stretchability, showing great potential for widespread use in wearables.

Enhancement in capacitance is crucial for the effectiveness of EAP-based strain sensors in real-world applications, such as health, activity monitoring, etc. [39,40,41], as explained below:

- Sensitivity and accuracy: A high capacitance means that the sensor can store more charge per unit voltage and, therefore, may be more responsive to small changes in strain. This increased responsiveness can indeed enable the sensor to detect subtle movements with greater accuracy, and consequently, the sensitivity of the sensor might also be improved.
- Wide Range of Applications: The enhanced capacitance in EAP strain sensors broadens their potential applications by enabling increased sensitivity, accuracy, and the ability to capture different types and levels of strains. This makes them versatile tools for a wide spectrum of industries and fields, from healthcare to aerospace, robotics, etc.
- Improved Signal-to-Noise Ratio: Higher capacitance results in a stronger signal relative to background noise. This means that the sensor can differentiate between the actual physiological signals and any interference or noise, leading to more reliable and trustworthy measurements.
- Miniaturization of Sensor Components: As capacitance is directly related to the geometry and dielectric properties of the material, an increase in capacitance can enable the design of smaller sensor components without compromising sensitivity. This is particularly important in the miniaturization of electronic devices, where space is often a critical factor.
- Reduced Interference: In real-world environments, sensors can be exposed to various types of electromagnetic interference. Higher capacitance can make the sensor less susceptible to such interference, resulting in more reliable and accurate readings.

As explained above, enhancement in capacitance is pivotal for EAP-based strain sensors in health monitoring applications, as it directly impacts their sensitivity, accuracy, etc. These factors are crucial for ensuring that the sensors provide meaningful and actionable data for a wide range of health-related measurements [42,43,44].

In this article, we enhanced the capacitance of EAP sensors by doping method. Doping in an Ionic Electroactive Polymer (EAP) strain sensor involves introducing additives into the material to alter its electronic properties. This process can have a significant impact on the sensor’s performance, including its capacitance. Doping can enhance capacitance as follows:

- Increase the ionic mobility: Doping can enhance the mobility of ions within the EAP material. This means that the ions can move more freely in response to an applied electric field. As a result, the material can respond more effectively, leading to higher capacitance.
- Reduce internal resistance: Doping can lower the internal resistance of the EAP material. This is crucial because a lower resistance allows more of the applied voltage to be utilized for inducing ion movement, rather than being lost as heat. With reduced resistive losses, the material can store more charge, resulting in increased capacitance.
- Improve dielectric properties: Doping can modify the dielectric properties of the EAP material, such as its permittivity. Materials with higher permittivity have a greater ability to store electric charge. Consequently, doping can lead to an increased capacity for charge storage and higher capacitance.
- Enhance surface charge density: Doping can lead to an increase in the surface charge density of the EAP material. This means that more charge can be stored at the material’s surface. Since capacitance is directly related to the amount of charge stored, this increase in surface charge density results in higher capacitance.
- Optimize ionic band structure: Doping can modify the ionic band structure of the EAP material. This impacts the energy levels available for electron movement, which, in turn, affects the material’s capacity to store charge. By tuning the band structure, doping can lead to improved charge storage and higher capacitance.
- Improve electrical conductivity: Doping can enhance the electrical conductivity of the EAP material. This improved conductivity facilitates a more efficient distribution of charge within the material, leading to higher capacitance.
- Controlled defects and dislocations: Doping can influence the presence and distribution of defects and dislocations in the crystal lattice of the EAP. These structural features can impact the material’s electronic properties and, consequently, its capacitance.

It is important to note that the specific effects of doping will depend on factors such as the choice of dopant, its concentration, and the characteristics of the EAP material. Additionally, the doping process should be carefully controlled to ensure that it enhances the desired properties without introducing undesirable side effects. Experimental testing and validation are crucial steps to assess the impact of doping on the capacitance of an ionic EAP strain sensor.

EAPs are typically made from organic polymers, such as plastics or rubbers, chemically modified to allow for electrical conductivity. The conductivity can be achieved through the incorporation of conductive particles, such as carbon or metals, into the polymer matrix [45,46,47,48]. Incorporating conductive particles into the polymer matrix is a fundamental method to enhance electrical conductivity in EAPs. These conductive particles play a pivotal role in facilitating the flow of ions throughout the material. This, in turn, enables the EAP sensor to respond dynamically to external stimuli. Such an improved composite film can potentially elevate the EAP sensor’s performance, broadening its applications from robotics to medical devices. Here, we present a highly elastic composite dielectric film that enhances the capacitance of the sensor.

When dodecyl-benzene sulfonic acid (DBSA) doped polyaniline and styrene-ethylene-butylene-styrene (SEBS) rubber composite film is sandwiched between carbon grease electrodes, it forms a device called an ionic type of electroactive polymer (EAP) strain sensor [49,50,51,52,53,54]. This type of sensor uses a composite film made of SEBS rubber and DBSA-doped polyaniline as the sensing element. The SEBS rubber provides mechanical stability and flexibility, while the DBSA-doped polyaniline contributes to the ionic conductivity of the composite film. The carbon grease electrodes serve as the stretchable electrical contacts for the sensor. When this type of sensor is subjected to an external stimulus, such as pressure or strain, the ionic conductivity of the composite film changes, which can be detected by an electronic device [55], this allows for the measurement of the external stimulus. This type of EAP sensor has high sensitivity and fast response times, making it useful in a variety of applications, such as medical devices and robotics. We have used carbon electrodes. Carbon-based materials can degrade over time due to environmental factors such as moisture, temperature changes, or mechanical stress. This degradation can lead to a decrease in sensor performance and reliability over time.

## 2. Experimental Section

### 2.1. Materials

Styrene–ethylene–butylene–styrene (SEBS) rubber was purchased from Sigma-Aldrich Co., USA [56] and DBSA-doped polyaniline was synthesized in our laboratory at Kyutech, Japan [57]. Carbon grease electrodes were purchased from MG Chemicals, Canada [58]. Conductive double-sided adhesive carbon tape was purchased from Nissin Co. Ltd., Japan [59]. An in-house uniaxial tensile system was used for the mechanical characterization of sensors. This system used a computer-controlled X-Y stage controller (Mark-102, Sigma Koki, Japan), load cell (LU-10 K, Kyowa, Japan), and digital Oscilloscope (TDS 2001C, Techtronic, USA).

### 2.2. Sensor Fabrication Process

The sensor is a three-layered device comprising a dielectric layer sandwiched between two conducting electrodes. This design provides the sensor with the ability to stretch and maintain its functionality. The sensor proposed in this article was fabricated using a composite film as a stretchable dielectric material and carbon grease as an electrode. Polystyrene-block-poly(ethylene-ran-butylene)-block-polystyrene-graft-maleic anhydride (SEBS-g-MA) and DBSA (dedocyl benzene sulfonate acid)-doped Polyaniline (PANI) were used as base materials for fabricating proposed sensor [60,61]. The stretchable composite film was fabricated through casting using toluene solutions. (at a given concentration) in a glass Petri dish. The detailed steps involved in the fabrication process for yielding DBSA-doped polyaniline salt and the composite film are detailed in the following subsections.

#### 2.2.1. DBSA-Doped Polyaniline Salt Yielding Procedure

1. Solution A—In a double jacket reaction vessel fitted cooling circulator and magnetic stirrer, add distilled Aniline (1.9 gm; 20 mmol), DBSA (6.8 gm; 30 mmol), and 50 mL solvent (Ethanol 30 mL and 20 mL distilled water solution).
2. Cool solution A at −5 °C and stir the solution for 30 min
3. Solution B—In another conical flask, dissolve Ammonium Peroxodisulfate (5.7 gm; 25 mmol) in 50 mL distilled water.
4. Add solution B into Solution A under continuous cooling and stirring; continue the polymerization reaction for about 6 h under this condition
5. Filter the polymer as a green precipitate at Buchner and wash with ample distilled water and methanol mixture; after drying, DBSA-doped polyaniline salt is ready for further use.

#### 2.2.2. Steps Involved in the Fabrication Process of Composite Film

1. Polystyrene-block-poly(ethylene-ranbutylene)-block-polystyrene-graft-maleic anhydride (SEBS-g-MA) and toluene are combined. The mixture is stirred at 1500 revolutions per minute (rpm) at 90 °C for 90 min. Keep the solution as it is at room temperature for 30 min.
2. DBSA-doped Polyaniline solution (20%) is added to the SEBS rubber solution
3. The solution is stirred again at 1500 rpm at 90 °C for 90 min (can be up to 2 h accordingly) and kept for 30 min at room temperature to ensure thorough mixing and uniform distribution of components.
4. The prepared solution is poured into a glass Petri dish in the desired shape at 90 °C for two hours (can be up to 3 h accordingly). The Petri dish is then left in an open environment at room temperature until the film cools down.
5. The resulting composite film is taken out and carbon grease is applied to both sides of the film. Carbon conducting tape is affixed to both sides to make the electrical connections.

#### 2.2.3. Schematic Diagram Representation of the Fabrication Process of the DBSA-Doped Sensor

**Figure 1:**
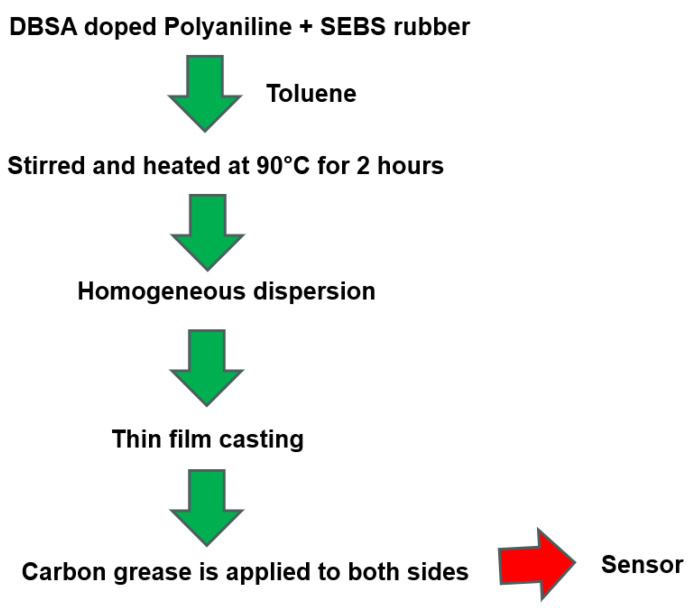
Schematic diagram of the steps involved in the fabrication process of the sensor.

The steps involved in the fabrication process of the DBSA-doped sensor can be understood from the schematic diagram as shown in Figure 1.

#### 2.2.4. SEM Characteristics

DBSA-doped composite film underwent microscopic surface analysis using a field emission scanning electron microscope (FE-SEM, JEOL, JSM-7800 F).

**Figure 2:**
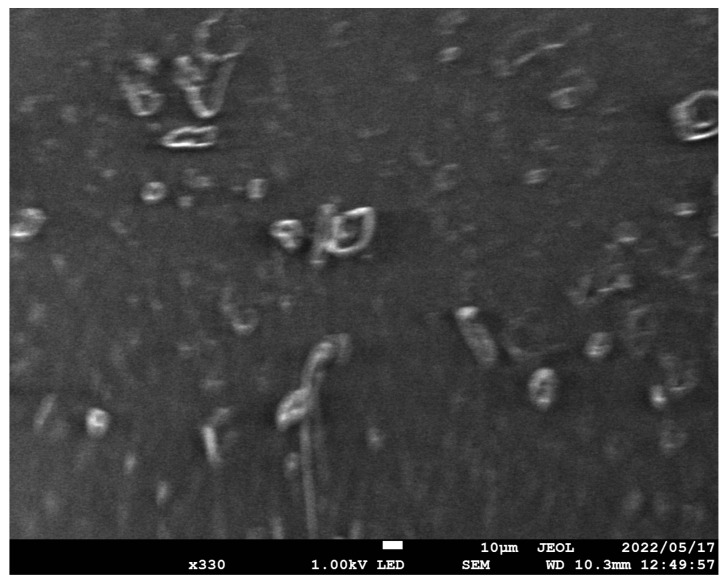
SEM characteristics of composite film.

A Scanning Electron Microscopy (SEM) image of DBSA-doped composite film can be seen in Figure 2.

#### 2.2.5. Block Diagram Representation of Three-Layer Sensor and DBSA-Doped Film

**Figure 3:**
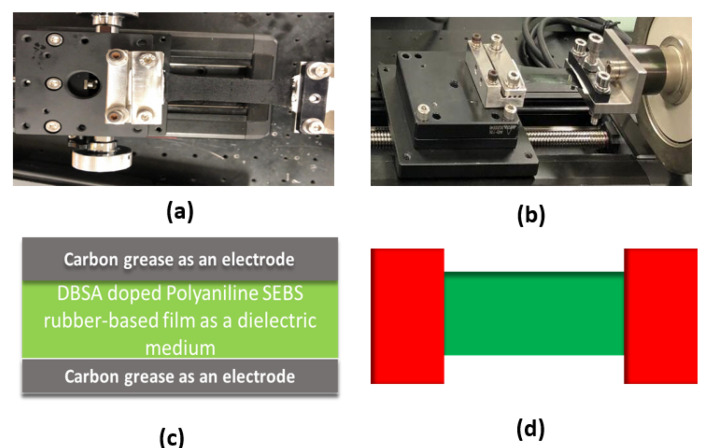
Actual Photograph of the sensor fixed between two jaws of the tensile machine (**a**). DBSA-doped polyaniline SEBS rubber-based dielectric film (**b**). Schematic representation of the three-layered sensor (**c**). Rectangular type of the specimen used in different experiments (**d**).

The block diagram of the three-layer sensor and DBSA-doped film is given below in Figure 3. Here, we can see that a rectangular specimen was used to measure the sensor’s capacitance and tensile properties. The green part shows the sensor’s active area and the red part on both ends is for gripping.

### 2.3. Capacitance Measurement

**Figure 4:**
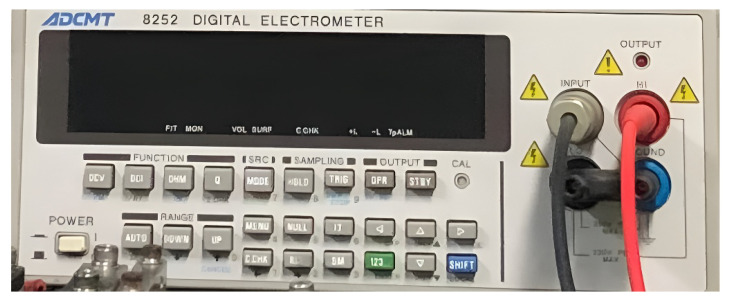
Digital electrometer for measuring capacitance.

A computer-controlled and programmable digital electrometer (Advantest ADCMT 8252) was used to measure the capacitance of strain sensors as shown in Figure 4. The positive and negative probes of the electrometer were connected to the conducting carbon tape connected to the upper and lower electrodes of the sensor. Once the connections are established, the electrometer applies a known voltage to the capacitor and measures the resulting charge. Through this process, the capacitance of the film capacitor is calculated based on the relationship between charge, voltage, and capacitance.

Capacitance was measured at the sensor’s 100% strain value using a movable, computer-controlled X-Y stage controller. For measuring capacitance rectangular type of specimen was used. The active length, width, and thickness of the sensor were 3 cm, 1.5 cm, and 0.05 mm, respectively. In all cases, the error was less than 10% for five samples.

### 2.4. Tensile Testing Measurements

Tensile testing of an Electroactive Polymer (EAP) strain sensor involves subjecting the sensor to controlled mechanical stress to assess its response to deformation. A rectangular type of strain sensor was fixed between both jaws of the uniaxial tensile machine for mechanical characterization, such as the stress-strain curve, elongation at break test, etc.

**Figure 5:**
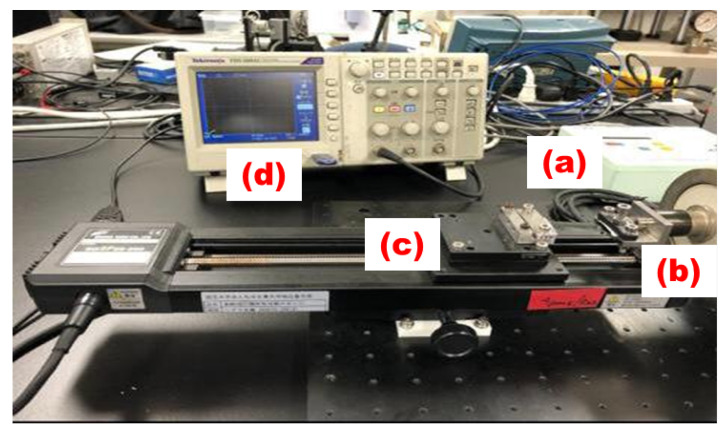
Stage controller (**a**) Load cell (**b**) Mobile stage (**c**) Digital oscilloscope (**d**).

Our tensile measurement system as shown in Figure 5 consists of stage controller (a), 100 Newton load cell (b), mobile stage (c), and a Digital oscilloscope (d), as explained below in detail.

(a) Stage Controller. The stage controller is a crucial element responsible for regulating the movement of the mobile stage. It allows precise control over parameters such as speed, distance, and direction during the tensile test. This ensures that the test is conducted under controlled conditions, allowing for accurate measurements.

(b) Load Cell. The load cell is a crucial component responsible for measuring the force applied to a specimen during a tensile test. It works on the principle of converting mechanical force into an electrical signal. In our tensile testing system, we used a 100 Newton load cell.

(c) Mobile Stage. The mobile stage is the component responsible for holding and moving the specimen during the tensile test. It allows controlled and uniform stretching or pulling of the material being tested. The stage controller governs the movement of this stage, ensuring that the test is conducted accurately and consistently.

(d) Digital Oscilloscope. The digital oscilloscope is a sophisticated instrument used for visualizing and analyzing electronic signals. In the context of a uniaxial tensile machine, it is employed to monitor the electrical signals generated by the load cell. This provides real-time data on the force applied to the specimen during the test, allowing for precise measurements and analysis.

In summary, the stage controller (a) regulates the movement of the mobile stage (c), the load cell (b) measures the force applied to the specimen, and the digital oscilloscope (d) assists in visualizing and analyzing the data produced by the load cell. Together, these components work in tandem to conduct accurate and controlled tensile tests on specimens.

Stress-strain, elongation at break, and relaxation tests were performed using this tensile test set up and controlled by a laptop using a LabView program. Tensile testing of an EAP (electroactive polymer) strain sensor involves applying a tensile force to the sensor and measuring its response. This is typically done by mounting the sensor in a tensile testing machine and applying a controlled tensile force to the sensor while measuring its output. During the testing, the sensor’s response to the applied stress or strain is recorded and analyzed to determine its stress-strain characteristics, which were calculated using the following relations:

(1) $$\sigma =\frac{F}{A}$$

(2) A=w·t

(3) $$\varepsilon =\frac{\Delta L}{L}$$

(4) λ=1+ε

where:

- σ is stress;
- ϵ is strain;
- ΔL is change in length;
- *L* is the initial length;
- λ is the extension coefficient;
- *A* is a cross-sectional area;
- *w* is the width of the specimen;
- *t* is the thickness of the sensor.

For measuring tensile characteristics, a rectangular type of specimen was used. The active length, width, and thickness for stress strain and stress relaxation tests were 2.5 cm, 1 cm, and 0.2 mm, respectively (green part only). The total length and width of the specimen were 5 cm and 2.5 cm, respectively (including clamping, i.e., the red part). The dimensions of the clamping part can be adjusted to ensure a good grip in the tensile machine’s jaw for fixing the specimen. In all cases, error was less than 10% for five samples and the median value was used for post-processing of data.

In our tensile testing system, the output data were recorded in volts (V) and time (s).

After that, we multiplied the voltage by a factor of 20 (calibration purpose) to calculate the force (according to the norms of the tensile machine; load cell). To determine the displacement, we multiplied time by speed. Later, we calculated the stress-strain curve using the specimen’s dimensions.

### 2.5. Sheet Resistivity Measurement

Sheet resistivity measurement of an Electroactive Polymer (EAP) strain sensor involves determining the electrical resistance across a known area of the sensor material. This measurement is critical in understanding the sensor’s electrical conductivity, which is a key factor in its performance. Sheet resistivity measurement of the sensor was conducted by using a four-point probe-based sheet resistivity meter.

A precision instrument, such as a four-point probe, is then used to apply a known voltage across the electrodes and measure the resulting current. By precisely controlling the geometry and the applied voltage, it is possible to calculate the sheet resistivity, which is expressed in ohms per square.

**Figure 6:**
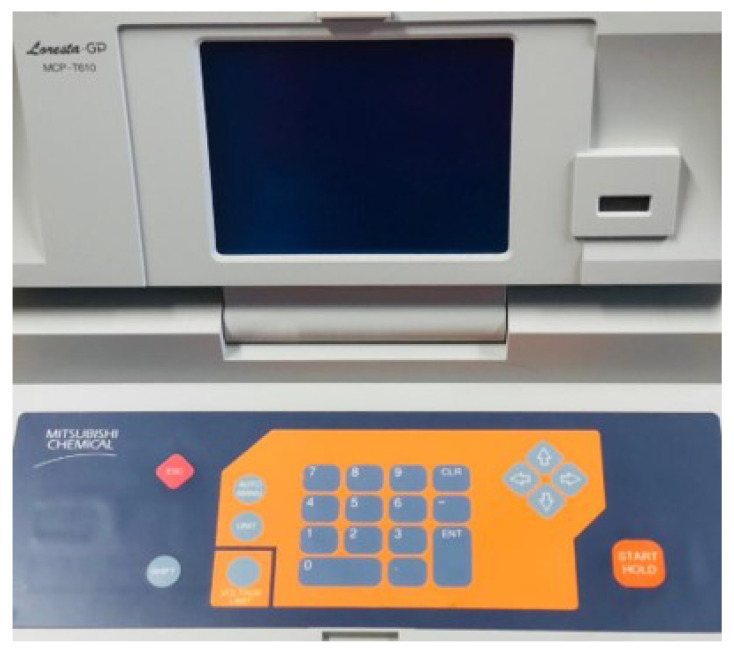
Sheet resistivity meter.

As shown in Figure 6, a four-point probe-based sheet resistivity meter uses four-point probes to measure the sheet resistivity of a sensor. The four-point probe consists of four electrical contacts placed at the corners of a square on the surface of the sheet.

## 3. Results and Discussion

### 3.1. Electrical Characterization

The electrical characterization of an Electroactive Polymer (EAP) strain sensor involves assessing its electrical properties to understand how they change in response to mechanical deformation. This process is crucial for evaluating the sensor’s performance and suitability for specific applications. Electroactive polymer-based strain sensors are three-layered devices. The upper and lower layer is made from compliant conducting electrodes and the middle part is made from the stretchable dielectric film [62].

**Figure 7:**
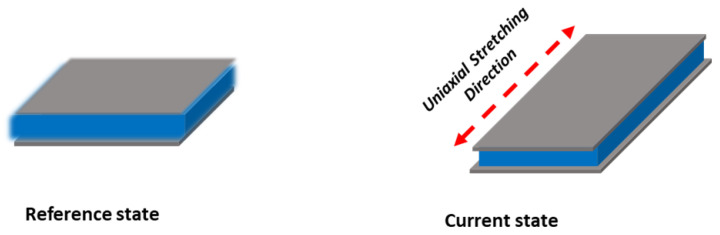
Basic principle of operation of the EAP-based strain sensor.

As shown in Figure 7, the gray part shows electrodes and the blue part shows dielectric film, and this arrangement is very similar to a parallel plate capacitor. When a uni-axial force is applied to the EAP strain sensor, the sensor deforms, which in turn changes the sensor’s capacitance.

The capacitance of this type of sensor depends upon the extent of the stretching and can be given by the below equation:

(5) $$C=\frac{\varepsilon_{r}\varepsilon_{0}A}{t}$$

where:

- $\varepsilon_{r}$ is the dielectric constant;
- $\varepsilon_{0}$ is the permittivity of free space;
- *A* is the active polymer area;
- *t* is the thickness of the dielectric film.

From the above equation, it can be seen that capacitance depends upon the specimen’s geometry and the dielectric constant of the dielectric (DE) film. We increased the dielectric constant of the dielectric film to enhance capacitance. We used a DBSA (dedocyl benzene sulfonate acid)-doped polyaniline (PANI) to increase the dielectric film’s dielectric constant in fabricating SEBS rubber-based composite film. DBSA is a dopant that can be used to increase the conductivity and the dielectric constant of PANI, a conducting polymer (CP).

By incorporating DBSA-doped PANI into the EAP material, the capacitance of the sensor can be significantly increased. The high conductivity of the PANI composite film enables fast ionic migration, which results in a significant change in electrical conductivity in response to mechanical strain (as shown in the below table), leading to a higher capacitance and sensitivity of the EAP strain sensor. As explained in the introduction section, doping also increases ion mobility, reduces resistance, and leads to improved charge storage capacity and higher capacitance. This method is a promising approach for enhancing the performance of EAP strain sensors for various applications.

In Table 1, measurements of the capacitance at 0% strain and sheet resistivity at the rest position are shown for both the SEBS rubber-based and DBSA-doped strain sensor. As shown in the table, we can conclude that doping enhanced the capacitance drastically.

**Figure 8:**
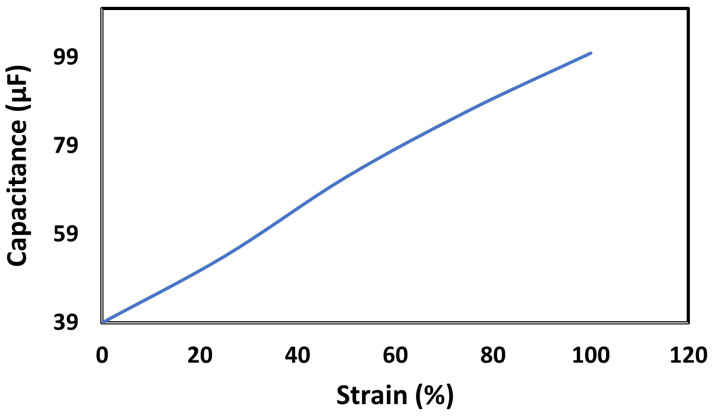
Capacitance of the DBSA-doped strain sensor at different strain values.

Figure 8 illustrates the corresponding capacitance values at various strain levels.

From the above figure, we can see that the capacitance of the doped sensor varies from 24.7 µF to 100 µF for a strain range from 0% to 100%.

To test the durability of this sensor, we calculated its capacitance after 1000 stretched and relaxed cycles at 100% strain value. The measured capacitance was 87 µF, which shows that this sensor has the potential to be widely used in wearables.

Generally, elastomeric films are used as a dielectric medium in capacitive-type soft strain sensors. These elastic films exhibit a sheet resistivity in the range of $10^{10}$ Ω/square [67,68]. However, after doping, we enhanced the conductivity of the dielectric film. The sheet resistivity of DBSA-doped film was measured sheet 3.7 × $10^{6}$ Ω/square. The enhancement in dielectric constant was achieved by incorporating a conducting polymer into the matrix elastomer backbone to form a composite film, which in turn enhanced the capacitance drastically. All capacitive sensors based on elastomers discussed in the introduction and listed in Table 1 exhibit maximum capacitance value below 2000 pF, even at the highest level of deformation consequently we can say doping has significantly enhanced the capacitance.

The Gauge factor (*GF*) or sensitivity (*S*) of such types of sensors can be defined by the equation given below:

(6) $$\;S=\frac{\Delta C}{C_{o}\cdot \varepsilon }$$

where:

- ΔC is change in capacitance;
- $C_{0}$ is the initial capacitance;
- ϵ is strain.

For DBSA-doped film, sensitivity was measured approximately 3 at 100% strain (ε=1).

### 3.2. Mechanical Characterization

Mechanical characterization of EAP-based strain sensors is critical for ensuring their optimal performance in various applications [69,70,71,72]. A comprehensive understanding of their mechanical behavior under different conditions enables engineers to design and implement these sensors effectively. Each testing method provides unique insights into the material’s properties, allowing for informed material selection and design choices. Additionally, it aids in improving the durability and reliability of EAP sensors, ultimately enhancing their functionality in real-world applications.

We used a uniaxial tensile machine (UTM) for the mechanical characterization of the sensor. The sensor was fixed between two Jaws of the tensile machine for estimation of different characteristics for the sensor like elongation at break, stress-strain curve, and stress relaxation test, as explained in the next section.

#### 3.2.1. Stress–Strain Characteristics

The stress–strain characteristics of an Electroactive Polymer (EAP) strain sensor depict how the material responds to mechanical forces. Understanding the stress–strain behavior of an EAP strain sensor is crucial for designing applications where the material experiences varying levels of stress and strain, ensuring its optimal and reliable performance in real-world scenarios [73,74,75].

When elastomers-based sensors are subjected to loading and unloading cycles at a given applied strain, they can experience a phenomenon known as “stress softening”, which is characterized by a decrease in stress with each cycle [76,77,78,79]. Stress softening occurs in elastomers because the polymer chains in the material are not perfectly elastic and do not return to their original shape and position when the stress is removed. This leads to a decrease in the material’s modulus and an increase in its deformation with each loading and unloading cycle. This can be a drawback for certain sensor applications because it can decrease the sensor’s sensitivity and accuracy over time. To check this, we run the tensile test for three cycles on the below-mentioned test protocol [80,81]:

- Strain = 100%;
- Strain rate = 1 mm/s;
- Specimen type- rectangular (length = 25 mm, Width = 10 mm, and thickness = 0.2 mm).

**Figure 9:**
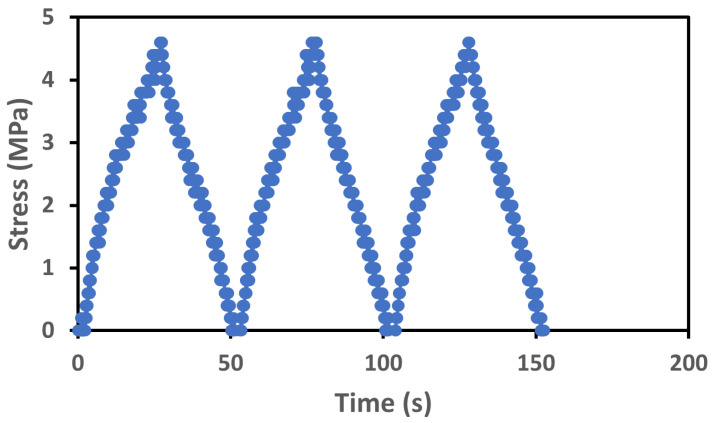
Stress–strain characteristics for three cycles.

From the Figure 9 illustrates the corresponding capacitance values at various strain levels, we can see that the sensor shows repetitive behavior and stress is almost constant in each cycle, so the stress-softening effect is negligible here. As mentioned earlier, the stress-softening effect refers to a decrease in mechanical stiffness or modulus of the material under sustained or cyclic loading conditions. In a strain sensor, this effect could lead to variations in the measured strain, as the material becomes progressively softer with repeated loading cycles. However, since the stress remains almost constant within our sensor within each cycle, it shows that the material maintains its mechanical properties consistently without significant softening. The absence of noticeable variations in the stress level throughout the cycles implies that the material’s stiffness or modulus is not changing significantly over time.

**Figure 10:**
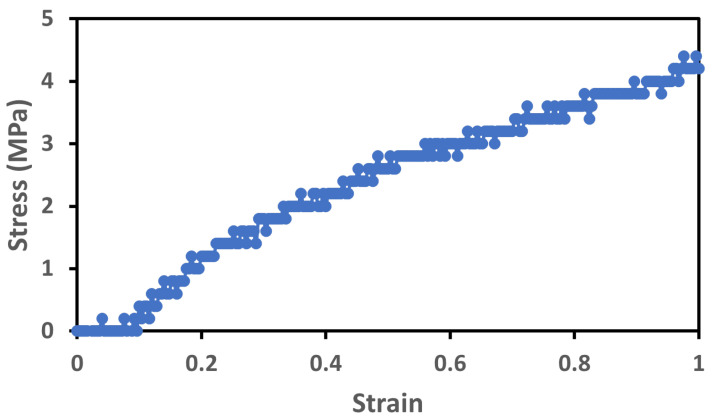
Stress–strain characteristics of the first loading cycle.

For the analysis of the stress–strain characteristics of the sensor, we can consider the first half of the loading cycle, as shown in Figure 10.

#### 3.2.2. Stress Relaxation Test

The stress relaxation test is a critical evaluation method used to understand how an Electroactive Polymer (EAP) strain sensor responds to sustained mechanical loads over time. In this test, a constant level of stress is applied to the material, and its corresponding strain is monitored as it evolves with time [82,83,84,85,86].

The primary goal of conducting a stress relaxation test on an EAP strain sensor is to gain insights into its long-term stability and durability under sustained loads. This is particularly relevant in applications where the sensor must maintain a specific shape or response over an extended period.

The stress relaxation test provides valuable data about the sensor’s long-term stability and durability. This information is essential for designing sensors that can maintain their performance characteristics over extended periods of use, which is particularly critical in remote health monitoring applications where continuous and accurate data are required.

The insights gained from a stress relaxation test can improve sensor design and material selection, ensuring that the EAP strain sensor meets the stringent requirements of real-world applications. This includes considerations for the material’s viscoelastic properties and its ability to maintain consistent performance under continuous load, which are pivotal in ensuring the accuracy and reliability of health-related data obtained from these sensors.

We used a uniaxial tensile machine for conducting a stress relaxation test. We fixed the sensor between two jaws of the tensile machine and stretched the sensor quickly (20 mm/s speed) until the value of strain was 100% and held the sensor at 100% strain value; furthermore, stress was measured as a function of time. The rectangular sample (length = 25 mm, Width = 10 mm, and thickness = 0.2 mm) sample was used for tensile testing.

**Figure 11:**
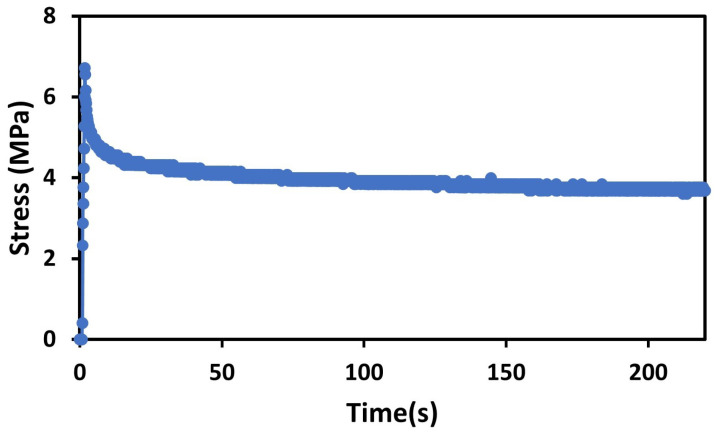
Stress relaxation characteristics.

From the Figure 11, it can be seen that stress becomes almost constant after a few seconds. Consequently, we can say that this sensor shows very small viscoelastic losses even at a large strain value. This characteristic is significant, as it implies that the material used in the sensor maintains its mechanical integrity and demonstrates a stable response over time. Viscoelastic materials possess both viscous and elastic properties, meaning they exhibit time-dependent deformation behavior and can dissipate energy during loading and unloading cycles. When subjected to constant strain or stress, a material with significant viscoelastic losses would experience a gradual decrease in stress over time, indicating energy dissipation within the material. The above graph shows that the stress remains nearly constant after a few seconds, even at a large applied strain. This behavior reflects that the strain sensor demonstrates very small viscoelastic losses and retains its mechanical properties over extended periods of strain application.

#### 3.2.3. Elongation at Break Test

The Elongation at break test is a crucial mechanical test conducted on Electroactive Polymer (EAP) strain sensors. It aims to determine the maximum amount of deformation or stretching the sensor can undergo before it ultimately fails or breaks. This test provides vital insights into the material’s ductility and its ability to withstand mechanical stresses. Engineers and researchers use this information to design and implement EAP strain sensors in a wide range of fields, including robotics, biomedical devices, and wearable technology. High elongation at break value indicates that the material is ductile and can withstand large deformation before breaking [87,88,89]. On the other hand, a low value indicates that the material is brittle and has a low resistance to deformation. We conducted an elongation at break test (speed was 1 mm/s) and clamped the sensor at both ends, and a tensile force was applied to the sample until it broke. A rectangular sample (length = 20 mm, Width = 12 mm, and thickness = 0.25 mm) was used for elongation at break test.

**Figure 12:**
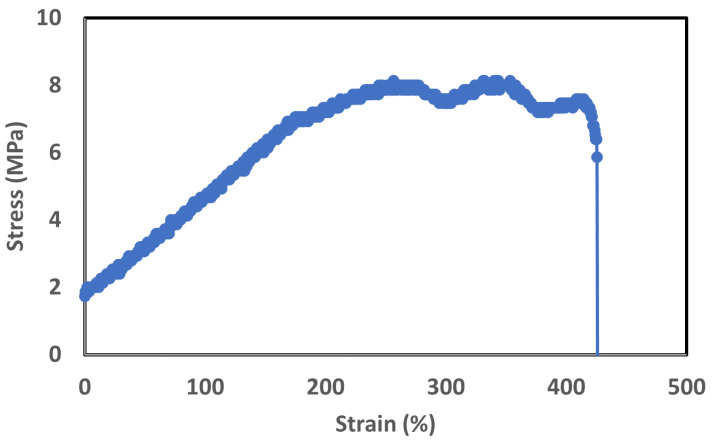
Elongation at break test.

From Figure 12, it can be seen that stress becomes almost constant after a few seconds. Consequently, we can say that this sensor shows very small viscoelastic losses even at a large strain value. This characteristic is significant, as it implies that the material used in the sensor maintains its mechanical integrity and demonstrates a stable response over time. Viscoelastic materials possess both viscous and elastic properties, meaning they exhibit time-dependent deformation behavior and can dissipate energy during loading and unloading cycles. When subjected to constant strain or stress, a material with significant viscoelastic losses would experience a gradual decrease in stress over time, indicating energy dissipation within the material. The above graph shows that the stress remains nearly constant after a few seconds, even at a large applied strain. This behavior reflects that the strain sensor demonstrates very small viscoelastic losses and retains its mechanical properties over extended periods of strain application, it can be seen that the sensor is more than 400% stretchable. The elongation at the break of an EAP strain sensor is an important consideration in applications where the material is subjected to high strains or mechanical stresses. It ensures that the sensor can withstand and recover from substantial deformations without permanent damage. Having a high elongation at break makes the strain sensor more resilient, providing reliable and accurate strain measurements over a wider range of deformations [90,91]. For our sensor, the maximum possible elongation stress is 8.1 MPa.

## 4. Conclusions

A highly stretchable and stable ionic EAP strain sensor, showing negligible viscoelastic losses, was fabricated. The results of the study demonstrate that incorporating DBSA into the polyaniline-SEBS rubber composite film can effectively enhance the capacitance of EAP-based strain sensors. It was observed that the sensor could be stretched up to 400% of the strain value, and drastic enhancement in capacitance was achieved. For strain levels ranging from 0 to 100%, the sensor exhibits capacitance values ranging from 24.7 to 100 µF, with a sensitivity measured 3 at the 100% strain level. This could have potential applications in various fields where reliable and efficient strain sensing is required, such as remote health monitoring, biomedical applications, or activity recognition.

## Figures and Tables

**Table 1:** Comparison between capacitance of undoped and doped sensors.

Dielectric Film	Electrode	Capacitance	Ref.
SEBS rubber	Carbon grease	32 pF	Our work
DBSA-doped	Carbon grease	24.7 µF	Our work
EPDM/carbon black nanoparticles	Fabric type	in pF range	[63]
Acrylic/silicone	Carbon based/thin metal film	in pF range	[64]
Ecoflex	silver nanowires	in pF range	[65]
Carbon filled elastomer	Composite	in pF range	[66]

## Data Availability

Data are available on genuine request.

## Abbreviations

The following abbreviations are used in this manuscript:

EAPs	Electroactive polymers
DBSA	Dodecyl benzene sulfonate anion
SEBS	Styrene-ethylene-butylene-styrene
PANI	Polyaniline
HMI	Human–machine interface
PDMS	Polydimethylsiloxane
UTM	Uniaxial tensile machine
DE film	Dielectric film
SS characteristics	Stress–strain characteristics
g-MA	Graft-maleic anhydride
CP	Conducting polymer
i-EAPs	Ionic electroactive polymers
e-EAPs	Electronic electroactive polymers
DEs	Dielectric elastomers
PVDF	Polyvinylidene fluoride
IPMC	Ionic polymer-metal composites
SEM	Scanning Electron Microscope
GF	Gauge factor
